# Bacteriology

**Published:** 1904-07-09

**Authors:** 


### PROGRESS IN MEDICINE AND SURGERY.

### BACTERIOLOGY.

**Diphtheria.**—Newman [1] in a note on the bacteriological diagnosis of diphtheria says that to establish the identity of the Klebs-Löffler bacillus, attention must be paid to the following four points :—(1) The macroscopic and microscopic appearance of the culture growth on blood-serum; (2) its staining properties, especially Neisser's reaction; (3) the formation of acid in neutral or slightly alkaline litmus-dextrine broth; (4) its pathogenicity, tested by inoculation in guinea-pigs. He quotes Graham Smith's statement that the Klebs-Löffler bacillus was found in 72 per cent. of 27,000 cases of certified diphtheria. In 12 metropolitan districts the Klebs-Löffler bacillus was found in 32·7 per cent. of all the swabs sent by medical men for examination; the figures for Liverpool were 29·3, and for Edinburgh 22·4. The number of these persons who have recently been in contact with diphtheria and who consequently harbour the bacillus in the throat or nose has been variously estimated. Brown visited 102 families in which the disease had occurred, and found the Klebs-Löffler bacillus in the swabbings from 32 apparently healthy persons, of whom 16 subsequently developed clinical signs of diphtheria. Probably not more than one or two per cent. of healthy persons harbour diphtheria bacillus in the throat or nose. A committee of the Massachusetts Association of Boards of Health investigated this last point in 1902 and found diphtheria bacilli in 3 per cent. of 4,250 healthy individuals. In 17 per cent. of these the bacilli were virulent. Salmon [2] investigated an outbreak of diphtheria at the Willard State Hospital and found undoubted diphtheria bacilli in 189 out of 1,423 well persons. He noted the extreme frequency of a diphtheria-like bacillus in the nose and throat of healthy persons, and to this bacillus he gave the title "type a." The morphological characters are as follows :—A thick bacillus composed of two deeply-stained segments, separated by an unstained portion. In shape it is an elongated ellipse, the spade-shaped segments joined base to base. Sometimes one segment is shorter and thicker than the other, or the two segments may be fused. In older cultures the staining is more polar in character, and generally the arrangement in films is more regular than is the case with diphtheria bacilli. True diphtheria bacilli resembling "type a" are not uncommon, but these show greater inequality of staining, and in the length of the segments, the ends are more pointed and the sides more concave. The "type a" bacillus is not virulent. It is found in from 25 to 60 per cent. of all noses and throats, and is commonly associated in the nose with staphylococci.

It seems possible that a way of distinguishing between true diphtheria bacilli and other forms may be found by means of the agglutinative reaction. Lipstein [3] immunised rabbits by the injection of diphtheria bacilli, and found that the serum not only had agglutinating properties, but that different varieties of diphtheria bacilli agglutinated with different degrees of readiness, so that it might be possible to say not only that the bacillus under examination was a diphtheria bacillus, but also to tell the variety. Louis Parkes' [4] report on the diphtheria outbreaks at the Chelsea Children's Hospital is interesting in this connection. In two and a-half years there had been eight outbreaks at the hospital, giving a total of 65 cases amongst the patients and nine cases amongst the staff. The precautions he recommended were:—(1) The prevention of infection from the out-patient department; (2) the disinfection of all discharges and secretions and the disinfection of the nurse's hands; (3) the disinfection of all feeding utensils; (4) bacteriological examination of the throat and nose of all cases admitted; (5) the prohibition of visiting; (6) sterilisation of milk; (7) no toys to be shared in common. Since adopting these measures the case rate of diphtheria in the wards has dropped from 31 per 1,000 admissions to 9 per 1,000 admissions. Eight hundred and fourteen cases were examined on admission. Of these 88, or 10·8 per cent., showed the presence of diphtheria bacilli; 162 were admitted with pseudo-diphtheria bacilli; and 563 gave negative results. Of those in the first class in whom the fauces and nose, though apparently healthy, yet contained diphtheria bacilli, 3·4 per cent. subsequently developed clinical diphtheria. Of the cases showing pseudo-diphtheria bacilli 1·9 per cent. developed the disease, and of those giving negative results 1·6 per cent. developed diphtheria. From this it would appear that the presence of pseudo-diphtheria bacilli in the nose and throat does not point to an increased risk of the subsequent development of diphtheria. Michelazzi[5] draws attention to a fact which is too often forgotten, namely, that there may be definite false membranes in the fauces, nose, or larynx caused by other organisms than the Klebs-Löffler bacillus. Of 37 cases clinically resembling diphtheria, 15 were found to contain diphtheria bacilli; 19 were due to cocci; two to Friedlander's pneumo-bacillus, and in one case no examination was made. This is the first occasion on which the pneumo-bacillus has been found in acute anginas, though it may be remembered that Leitz described a case as due to the B. Coli. He regards the infections by cocci as being even more dangerous than those due to the Klebs-Löffler bacillus, as these are uninfluenced by diphtheria antitoxin, and there is greater danger of pulmonary complication. It is a mistake to suppose that a membranous affection confined to the larynx is necessarily diphtheritic, as many of the most dangerous cases are purely coccal in origin. In the course of examinations of cows' milk Bergey[6] has detected pseudo-diphtheria bacilli associated with staphylococci or streptococci. Such milk not uncommonly was found to contain pus. He classes these bacteria into three groups. Firstly, those which in morphologic and biologic characters resemble the Klebs-Löffler bacillus. Secondly, those which morphologically resemble the diphtheria bacillus, but which differ in having much freer growth on culture media and in forming a yellow or orange pigment. Thirdly, those whose morphologic character are only remotely suggestive of the diphtheria organism, and which produce alkali in milk and indol in broth. These last show Neisser's polar granules. None show pathogenic properties. Probably they are derived accidentally from the skin and mucous surfaces of cattle, but in Bergey's opinion they are related biologically to the true diphtheria bacillus. The importance of a knowledge that these organisms may exist in milk is obvious, and might prevent a too hasty conclusion than an organism morphologically and culturally resembling the Klebs-Löffler bacillus was the exciting cause of an epidemic of diphtheria.

1 Practit., Nov. 1903. 2 Amer. Jour. Med. Sci., Jan. 1904. 3 Centrab. f. Bakt., Jena. 1903. 4 Trans. Epidem. Soc., Lond. vol. xxii. 5 Il Policlin, Feb. 1904. 6 Jour. Med. Research, May 1904.

*(To be continued.)*

